# Nischarin Is Differentially Expressed in Rat Brain and Regulates Neuronal Migration

**DOI:** 10.1371/journal.pone.0054563

**Published:** 2013-01-16

**Authors:** Yuemin Ding, Ruyi Zhang, Kena Zhang, Xinyou Lv, Yanan Chen, Aiqing Li, Linlin Wang, Xiong Zhang, Qiang Xia

**Affiliations:** 1 Department of Physiology, Zhejiang University School of Medicine, Hangzhou, China; 2 School of Medicine, Zhejiang University City College, Hangzhou, China; 3 Department of Neurobiology, Zhejiang University School of Medicine, Hangzhou, China; 4 College of Life and Environmental Sciences, Hangzhou Normal University, Hangzhou, China; 5 Gastroenterology Laboratory, Sir Run Run Shaw Hospital, Zhejiang University School of Medicine, Hangzhou, China; Universidade Federal do ABC, Brazil

## Abstract

Nischarin is a protein known to inhibit breast cancer cell motility by regulating the signaling of the Rho GTPase family. However, little is known about its location and function in the nervous system. The aim of the present study was to investigate the regional and cellular expression and functions of Nischarin in the adult rodent brain. As assessed by real-time PCR, Western blot analysis and immunostaining, we found that Nischarin was widely distributed throughout the brain, with a higher expression in the cerebral cortex and hippocampus. Double-labeling showed that Nischarin was expressed in neurons and was mainly located in the perinuclear region and F-actin-rich protrusions. The expression pattern of Nischarin in the brain was thought to be closely associated with its function. This was verified by our findings from cell migration assays that Nischarin regulated neuronal migration. These results provide a preliminary survey of the distribution of Nischarin in different regions and cell types in the rat brain. This might help to elucidate its physiological roles, and to evaluate its potential clinical implications.

## Introduction

Nischarin was first cloned from a mouse embryonic cDNA library and named by Alahari in 2000 [1]. In mouse Nischarin gene is found on chromosome 14, whereas in rat it is found on chromosome 16. The homology on the amino acid between mouse and rat is as much as 93%, suggesting the functional homology between the two isoforms. Nischarin interacts directly with integrin α5 and inhibits cell motility by affecting the signaling of the cytoskeleton regulated by Rho GTPases [2], [3], [4], [5]. It inhibits the Rac1 signaling pathway by p21-activated kinase (PAK)-dependent and PAK-independent mechanisms [6], and negatively modulates the LIMK/cofilin pathway [5], resulting in decreased cell motility. Recently, Nischarin was reported to be a suppressor in breast cancer progression by regulating integrin α5 expression and the downstream signaling, including FAK phosphorylation and Rac activation [7].

The Rho GTPase family plays important roles in many cellular functions, such as proliferation, apoptosis, migration, and gene expression, by regulating actin dynamics [8], [9]. In the nervous system, members of the Rho GTPase family are also actively involved in vital cellular processes, including morphological plasticity during neuronal polarization, migration and division [10], the formation of growth cones during axonal generation or regeneration [11], [12], [13], malformation of dendritic spines in mental retardation [14], [15], [16], and the development of brain tumors [9], [17]. Any factor in the nervous system that regulates the downstream signaling cascade of Rho GTPase is likely to contribute to the above processes.

Nischarin inhibits cancer cell migration by regulating the Rac1-PAK-LIMK pathway, one of the key downstream pathways of Rho GTPase [2], [6]. Thus, Nischarin is a possible candidate to play a crucial role in the brain. However, little is known about the distribution and function of Nischarin in the nervous system.

Therefore, we systematically investigated the localization of Nischarin at the regional and cellular levels in the adult rodent brain and assessed its effects on the motility of neuronal cells.

## Materials and Methods

### Ethics statement

The experimental procedures were approved by the Animal Ethics Committee of Zhejiang University and were carried out in accordance with institutional guidelines. All efforts were made to minimize the number of animals used and their suffering.

### Animals and cell lines

Male Sprague-Dawley rats weighing ∼250 g were purchased from the Experimental Animal Centre of Zhejiang University (Hangzhou, China). The mouse neuroblastoma (Neuro-2a) and rat adrenal medulla pheochromocytoma cell lines (PC-12) were purchased from the Shanghai Cell Resource Center, Chinese Academy of Sciences (Shanghai, China). Neuro-2a cells were cultured in Dulbecco's modified Eagle's medium (DMEM; Keyi, Hangzhou, China) supplemented with 10% heat-inactivated fetal bovine serum (FBS; HyClone, Logan, UT, USA) and 1% v/v penicillin/streptomycin (Sigma-Aldrich, St. Louis, MO, USA) in a 5% CO_2_ humidified atmosphere at 37°C. PC-12 cells were grown in RPMI 1640 medium (Keyi, Hangzhou, China) supplemented with 10% FBS.

### Quantitative real-time RT-PCR

Rats were anesthetized with 10% chloral hydrate (400 mg/kg) before decapitation. Tissues from different organs and different regions of the brain were immediately isolated on ice. Total RNA was extracted with TRIzol reagent (Invitrogen, Carlsbad, CA, USA) according to the manufacturer's protocol. cDNA was synthesized using a PrimeScript First-Strand cDNA synthesis kit (Takara, Dalian, China). Specific primers for Nischarin and GAPDH were designed using Primer Premier 5.0 software as follows: Nischarin primers, sense 5′-ACCTGCAGTCAGTCAACGTC-3′ and antisense 5′-CAGGAAGCAGTGTGTCAGGT-3′; GAPDH primers, sense 5′-TGATTCTACCCACGGCAAGTT-3′ and antisense 5′-TGATGGGTTTCCCATTGATGA-3′. Each 25-μl PCR mixture contained 12.5 μl SYBR Premix (Takara), 400 nM primers, and ∼10 ng template. All reactions were performed in triplicate. Thermal cycling was performed in a CFX 96 Real-Time PCR Detection System (Bio-Red, Hercules, CA, USA). Expression of Nischarin was assessed using the 2^−ΔΔCT^ formula.

### Protein extraction and Western blot

Proteins from different organs and brain regions were extracted with RIPA buffer (Beyotime, Shanghai, China) containing a protease inhibitor cocktail (Sigma). Tissue lysates were separated by SDS-PAGE and transferred to a PVDF membrane. After 2 h of blocking in 5% nonfat milk, blots were incubated overnight in primary antibodies (Nischarin, 1∶1000, BD Biosciences, San Jose, CA, USA; Integrin α5, 1∶1000, Cell Signaling Technology, Danvers, MA, USA; GAPDH, 1∶500, Good Here, Hangzhou, China) at 4°C, washed in TBS-Tween 20, and incubated in secondary antibodies for 2 h at room temperature. The protein bands were visualized by enhanced chemiluminescence reagents (Amersham, Arlington Heights, IL, USA) and were exposed to X-ray film. Quantification of band intensity was performed using NIH ImageJ software.

### Single-staining in sections

Rats were anesthetized and transcardially perfused with physiological saline, followed by 4% paraformaldehyde in 0.1 M phosphate buffer (pH 7.4). Brains were post-fixed in 4% paraformaldehyde for 1 day, and kept in sucrose solutions of increasing concentration (10, 20 and 30%) in phosphate-buffered saline (PBS) until they sank. Sections (40 μm) were prepared on a cryostat (Thermo Scientific Microm HM550), blocked with 10% goat serum for 2 h, incubated with primary antibody (mouse anti-Nischarin monoclonal antibody, 1∶100) overnight at 4°C, and then incubated for 2 h with secondary antibody anti-mouse FITC (1∶100) at room temperature, washing with PBS after each step. Negative-control staining was performed by the same procedure with the exception of primary antibody incubation. The fluorescent signals were examined using an Olympus FluoView FV1000 confocal laser scanning microscope. For analyze of Nischarin expression, fluorescent intensity was quantified by measuring intensity in tissues using ImageJ. Data were collected from five sections of each sample, and three samples were used.

### Double-staining in cell cultures

Neuro-2a and PC-12 cells were fixed in 4% paraformaldehyde for 10 min and processed with the basic procedures described above. Briefly, cells were permeabilized with 0.3% Triton X-100, blocked with 10% goat serum, and incubated with mouse anti-Nischarin monoclonal antibody (1∶100) and rabbit anti-Map-2 polyclonal antibody (1∶100) overnight at 4°C, followed by incubation with anti-mouse FITC (1∶100) and anti-rabbit Cy3 (1∶100) for 2 h at room temperature. After rinsing with PBS, coverslips were mounted onto the slides with a fluorescent mounting medium containing 4′, 6-diamidino-2-phenylindole (DAPI) to counterstain cell nuclei and imaged using an Olympus FluoView FV1000 confocal laser scanning microscope. The co-localization of Nischarin with Map-2 was performed using ImageJ.

### siRNA knockdown of Nischarin

A commercially-available siRNA containing a pool of three target-specific siRNA sequences of rat Nischarin (Santa Cruz, CA, USA) was transfected into Neuro-2a and MCF-7 cells using Lipofectamine 2000 reagent (Invitrogen), following the manufacturer's instructions to knockdown Nischarin mRNA. Control cell cultures were transfected with Lipofectamine alone and a non-specific siRNA (Santa Cruz). After transfection for 6 h, the transfection medium was replaced with complete medium and cells were incubated for a further 42 h before cell lysis and Western blot. Knockdown experiments were performed in duplicate on three separate occasions.

### Cell Proliferation Assay

The effect of Nischarin on the growth of Neuro-2a cells was determined using the MTT (3-[4,5-dimethylthiazol-2yl]-2,5-diphenyltetrazolium bromide) assay as previously described [18]. Briefly, cells were seeded in 200 µl DMEM on 96-well plates (1×10^5^/well), and transiently transfected with Nischarin siRNA or control siRNA. At 0, 12, 24 and 48 h after transfection, 100 µl MTT (5 mg/ml, Sigma) was added respectively. After a 4-h incubation at 37°C, 100 µl dimethyl sulfoxide (DMSO) was added to dissolve the formazan crystals. Cell viability was determined by measuring the absorbence at 570 nm using a microplate reader (Bio-Rad).

### Wound-healing scratch assays

Scratch assays were performed as previously described [19]. Briefly, PC-12 cells were seeded in 6-well plates at 5×10^5^ cells/ml and transiently transfected with Nischarin siRNA or control siRNA. At 48 h after transfection, a scratch was made down the center of each well using a plastic pipette tip. Along the scratch line, the detached cells and debris were gently washed away with PBS and the medium was replaced with serum-free culture medium. Cell migration was monitored every 12 h for 48 h under an inverted phase-contrast microscope (Nikon). At each time point, the cell migration edge was traced, and the distance to the initial scratch edge was measured using ImageJ. For each well, three different fields along the scratch were analyzed in triplicate. Motility was calculated as the percentage of the cell migration distance with respect to the initial scratch distance.

### Transwell cell migration assays

Transwell cell migration assays were performed as described elsewhere [4], [5], [20]. Briefly, the outside membrane of the transwell was coated with fibronectin. At 48 h after transfection with Nischarin siRNA or control siRNA, PC-12 or Neuro-2a cells were resuspended in serum-free medium at a density of 5×10^5^ cells/ml and seeded into the upper chamber. RPMI 1640 or DMEM containing 20% FBS was placed in the lower chamber. After incubation for 24 h at 37°C, the membranes of the transwells were removed and stained with DAPI. The number of migratory cells was counted five times in random fields under an immunofluorescence microscope. Experiments were performed in triplicate.

### Statistics

Data are presented as mean ± standard deviation. Unless stated otherwise, one-way analysis of variance (ANOVA) with Student's-Newman-Keuls test were used for statistical comparison when appropriate. Differences were considered statistically significant at p<0.05.

## Results

### Tissue distribution of Nischarin in the adult rat

**Figure 1:**
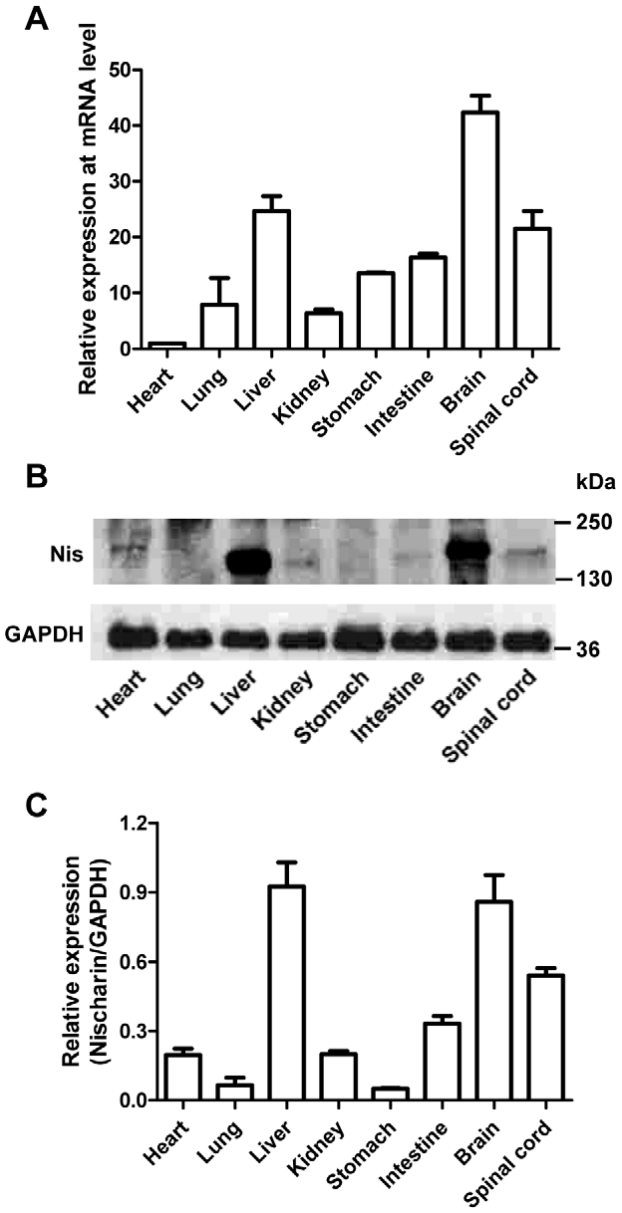
Nischarin is highly expressed in the brain of adult rats. (**A**) Total mRNA extracted from heart, lung, liver, kidney, stomach, small intestine, brain and spinal cord of adult rats was assayed by quantitative real-time PCR (n = 5). Relative quantification was assessed by normalizing the amount of Nischarin to the housekeeping gene GAPDH. (**B, C**) Protein samples from different tissues were analyzed by Western blot and the quantitative analysis was performed by normalizing the intensities of the hybridization signals to GAPDH (n = 5). Nischarin is highly expressed in liver, brain and spinal cord at both the mRNA and protein levels. Data are presented as mean ± SD.

To determine the regional distribution of Nischarin, real-time PCR was performed to quantify the pattern of Nischarin mRNA expression in adult rat tissues (heart, lung, liver, kidney, stomach, small intestine, brain and spinal cord). The results showed an ubiquitous expression pattern, with higher levels in the brain, spinal cord and liver (Fig. 1A). To confirm these results, Western blot analysis was then conducted to examine the expression of Nischarin protein with GAPDH as a control (Fig. 1B). Quantitative immunoblot analysis showed that Nischarin protein was expressed in all tissues, with higher levels in the liver, brain and spinal cord (Fig. 1C).

### Regional distribution of Nischarin in the rat brain

**Figure 2:**
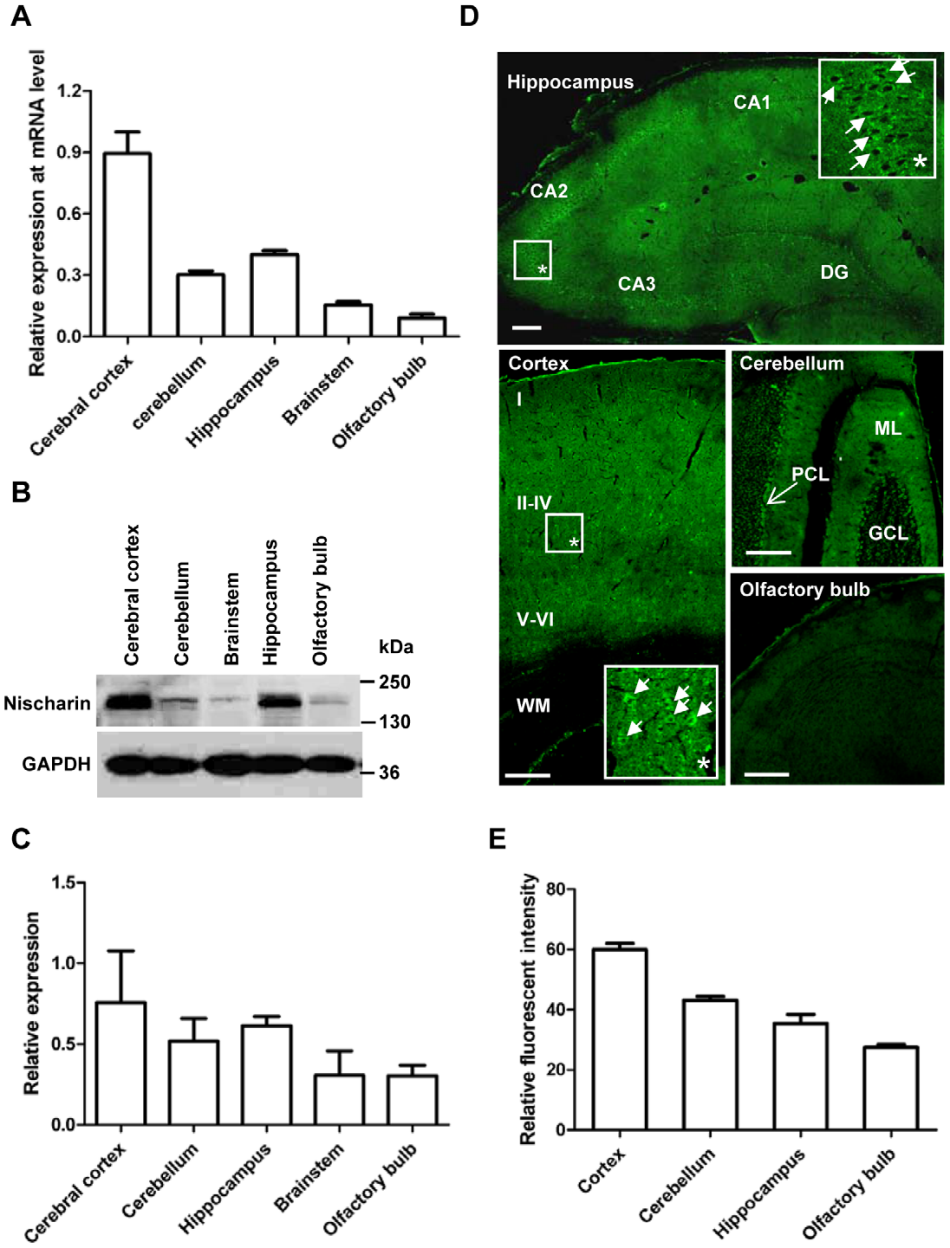
Regional distribution of Nischarin in the rat brain. The highest expression level of Nischarin mRNA was in the cortex, based on real-time PCR (**A**). This was confirmed by Western blot analysis and immunostaining data at the protein level (**B, C, E**). Data are presented as mean ± SD. n = 5. The overview immunofluorescence images and the higher magnification images (*) revealed that Nischarin was clearly present in the pyramidal neurons of the cerebral cortex, the CA subfields of the hippocampus, and the Purkinje cells in the cerebellum (**D**). DG, dentate gyrus; ML, molecular layer; PCL, Purkinje cell layer; GCL, granule cell layer; WM, white matter. Scale bars, 200 μm. Images are representative of 3 rats.

In order to determine the more detailed regional distribution pattern of Nischarin in the brain, real-time PCR and Western blot were performed on the cerebral cortex, cerebellum, hippocampus, brainstem and olfactory bulb (Fig. 2A). The highest expression level was in the cerebral cortex and hippocampus, while it was lower in the brainstem and olfactory bulb. Western blot confirmed that stronger bands were found from lysates of cortex and hippocampus (Fig. 2B), which was further demonstrated by quantitative immunoblot analysis (Fig. 2C). Immunofluorescence was conducted to determine the Nischarin protein expression in more detail (Fig. 2D). Nischarin signals were detected in the hippocampus, especially in the CA1, CA2 and CA3 regions, representing the pyramidal neurons. Interestingly, few labeling was observed in the hippocampal dentate gyrus (DG) granule neurons. In the cerebral cortex, Nischarin signals were located in the grey matter, but not in the white matter. Nischarin was expressed by neurons of all six cortical layers, with higher expression in layers IV-V pyramidal neurons. Moreover, both Purkinje cells and cells in the molecular layer of the cerebellum appeared to specifically stain with the Nischarin antibody, and the former showed a stronger signal. Somewhat weaker fluorescent signals were also exhibited in olfactory cells. In agreement with our real-time PCR and Western blot data, quantitative analysis of the fluorescent intensity further confirmed that the highest expression of Nischarin occurred in the cerebral cortex (Fig. 2E).

### Subcellular expression pattern of Nischarin in neuronal cell lines

**Figure 3:**
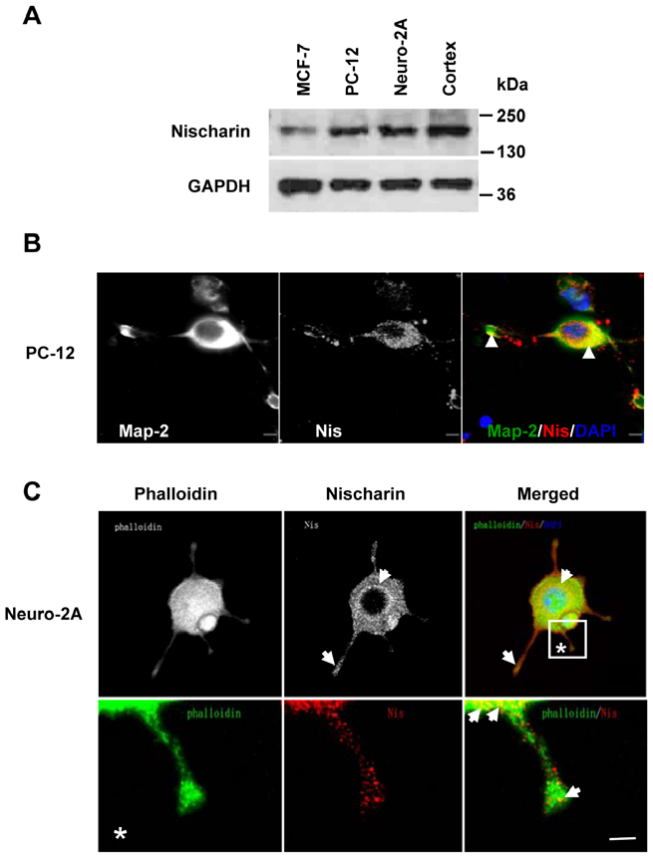
Subcellular expression pattern of Nischarin in neuronal cell lines. (**A**) Western blot data revealed the expression of Nischarin protein in both PC-12 and Neuro-2a cells. MCF-7 cells and cortical tissue lysates were used as the positive controls (n = 5). (**B**) Immunofluorescent double-staining was performed to reveal the subcellular distribution of Nischarin in PC-12 cells. Co-localization of Nischarin (red) and Map-2 (green) was observed in the cytoplasm and dendrite-like projections (arrowheads in B). (**C**) The subcellular distribution of Nischarin was further confirmed by staining Neuro-2a cells with Nischarin antibody (red) and FITC-phalloidin (green). Strong staining for Nischarin in the perinuclear region and F-actin-rich protrusions (arrows in C) was observed on the overview and the higher magnification images (*). Scale bars, 5 μm.

To investigate the expression of Nischarin in neuronal cell lines, we next performed Western blot analysis of total whole-cell lysates extracted from PC-12 and Neuro-2a cells. Positive controls consisted of total cell extracts from breast cancer MCF-7 cells and cerebral cortex tissue, which exhibited specific bands for Nischarin. Specific bands were found in blots of both PC-12 and Neuro-2a lysates (Fig. 3A), confirming the presence of Nischarin protein in these cells. To further explore the subcellular localization of Nischarin, we double-labeled PC-12 cells with antibodies raised against the neuronal marker Map-2 and against Nischarin. Co-localization of Nischarin and Map-2 immunoreactivity was found (Fig. 3B), showing that Nischarin was mainly expressed in the neuronal cell body and Map-2-positive dendrites. We have previously shown that, in MCF-7 cells, Nischarin regulates F-actin organization through the downstream cascade of the Rho GTPase family [5]. However, no studies have been conducted to address whether Nischarin is associated with actin structures in neuronal cells. Therefore, Neuro-2a cells were double-labeled with Nischarin antibody and FITC-phalloidin. Structures with F-actin were visualized by confocal microscopy which showed that Nischarin was expressed in a punctate pattern in the perinuclear region in the cytoplasm. Co-localization of Nischarin with F-actin was detected in both the cytoplasm and F-actin-rich filopodia-like protrusions (Fig. 3C). Higher-power images of cell protrusions revealed that Nischarin overlapped with F-actin at the leading edge of the protrusion (Fig. 3C).

### Nischarin regulates neuronal migration

**Figure 4:**
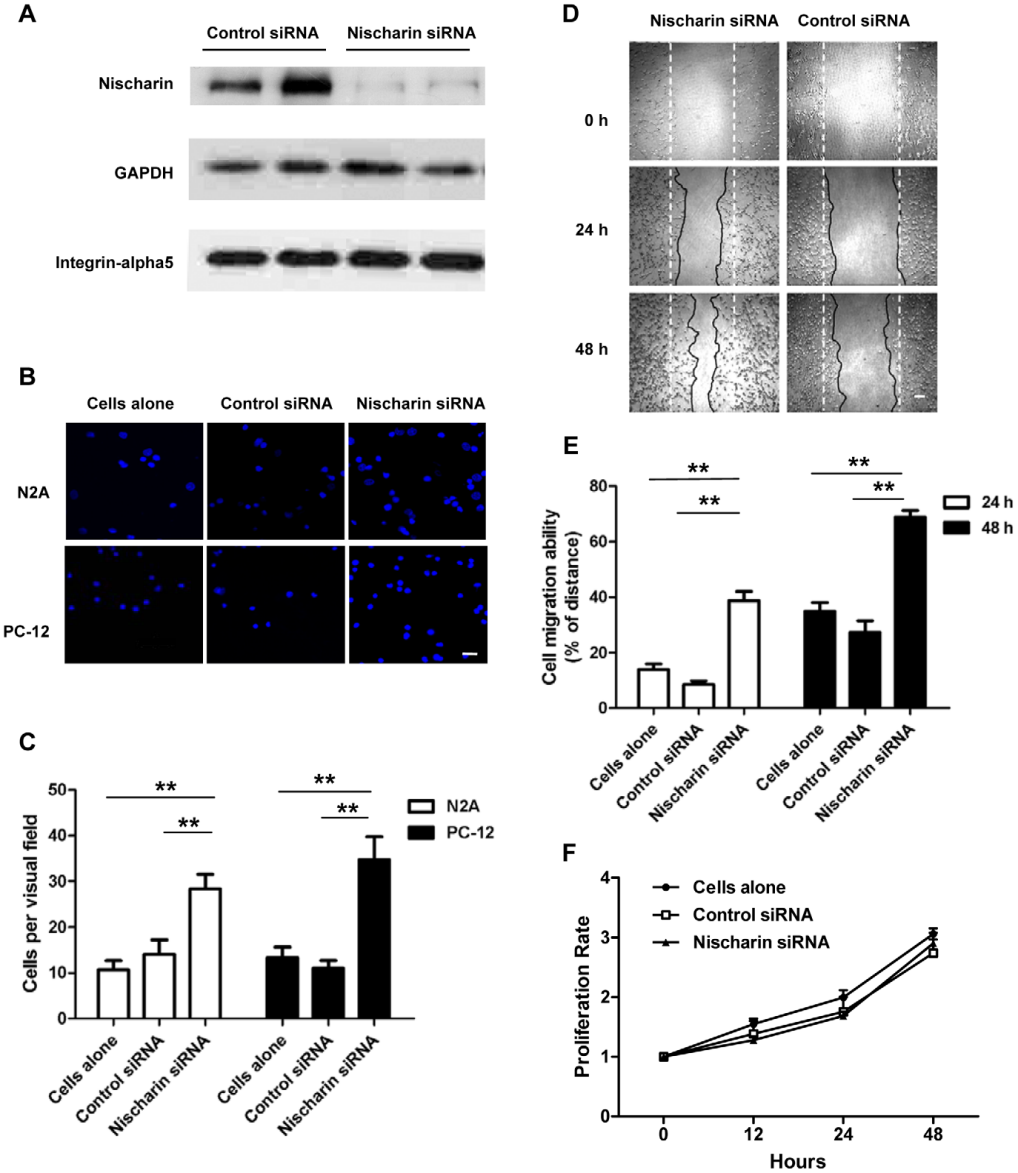
Knockdown of endogenous Nischarin promotes cell migration. PC-12 and Neuro-2a cells were transfected with anti-Nischarin siRNA or control siRNA. (**A**) Immunoblot data showed that expression of endogenous Nischarin, but not that of integrin α5 was remarkably reduced at 48 h after transfection in Neuro-2a cells. (**B**) Cells migrating across the membrane of the transwell were stained with DAPI. Scale bar, 20 μm. (**D**) Images of migrated cells subjected to scratch assays. Scale bar, 100 μm. The dotted straight lines indicate the dimensions of the scratch, and the solid irregular lines indicate the cell edges. (**C, E**) Quantitative measurements of the motility indicated enhanced migration in cells transfected with anti-Nischarin siRNA compared with the control siRNA. (F) Proliferation rates of Neuro-2a cells are determined using MTT assay over 48 h. Data are presented as mean ± SD. n = 9/group. One-way ANOVA. *p<0.05, **p<0.01.

The finding of intensive Nischarin fluorescence at the leading edge of protrusions suggested that it might be involved in neuronal migration. To verify the role of Nischarin in cell motility, anti-Nischarin siRNA or control siRNA was transfected into neuronal cells and migration assays were performed. The siRNA silencing efficiency was evaluated at 48 h after transfection by Western blot analysis (Fig. 4). We obtained efficient knockdown of endogenous Nischarin protein in Neuro-2a cells without significant influence on integrin α5 expression (Fig. 4A). Transwell migration assays revealed that anti-Nischarin siRNA induced significant increases in migration compared with the control siRNA in both PC-12 and Neuro-2a cells (Fig. 4B and 4C). These findings were further confirmed by wound healing scratch assays. Consistent with these results, anti-Nischarin siRNA remarkably promoted PC-12 cell migration at 24 h and 48 h after transfection (Fig. 4D and 4E). Furthermore, the increased cell motility induced by siRNA transfection was not dependent on cell proliferation (Fig. 4F).

## Discussion

Determining the regional, cellular and subcellular distribution is a basic step for better understanding the physiological function of a specific protein. Here, we systematically investigated the localization of Nischarin at the regional and cellular levels in the adult rodent brain. Our results showed that the Nischarin protein and its mRNA were both widely distributed in the adult rat, but were enriched in the brain. These findings are in agreement with previously published localizations in the mouse and rat brain [1], [21], [22], although these reports only detected the expression of Nischarin at the mRNA level, and did not further investigate its presence in multiple regions of the brain. In addition, using immunofluorescence analysis, we found that the strongest staining for Nischarin was present in the pyramidal neurons of the cerebral cortex and hippocampal CA regions, and Purkinje cells of the cerebellum, while few labeling in the hippocampal DG region and olfactory bulb.

We further explored the subcellular distribution of Nischarin in rat pheochromocytoma PC-12 and mouse neuroblastoma Neuro-2a. We found intense immunostaining of cell bodies in a punctate pattern in the perinuclear region in both cell types, in accord with the previous work by Alahari and coworkers [1]. In addition, strong staining for Nischarin was found at the leading edge of F-actin-rich filopodia-like protrusions, which has not been described before.

Based on the finding that Nischarin is strongly expressed in a wide variety of brain regions and cells, we asked what specific functions Nischarin might perform in the nervous system. To address this question, we investigated the migration of neuronal cells by siRNA knockdown of the endogenous expression of Nischarin. We found that silencing Nischarin greatly promoted the motility of both rat and mouse derived neuronal cells, indicating that it is a negative regulator in neuronal migration. This is comparable to our previous studies of breast cancer cells [5]. However, further studies are needed to determine whether Nischarin inhibits neuronal migration through a signaling pathway involving the Rho GTPase family.

Neuronal migration plays a central role in the formation of the brain during the embryonic period. For instance, the migration of neurons results in the formation of an orderly 6-layered structure during the development of neocortex [23]. The early-born and mature neurons form the inner layers of cortex, while the later-born neurons form the out layers. Our Immunofluorescence data showed a higher expression of Nischarin in layers IV-V of cortex, indicating that Nischarin is specific expressed by the mature neurons which have reached their final destination and stopped migration. It is also reported that a significant number of neurons migrate after birth and persist into adulthood [24]. Neural stem cells exist in the subventricular zone (SVZ) and the hippocampal DG region and migrate toward the olfactory bulb and granular cell layer of the DG [25], where few Nischarin labeling was observed in our experiments. This is not difficult to understand that the absence of Nischarin in the newborn neurons enables them to move across the brain to reach their final destination, since Nischarin is found to be an inhibitory regulator in neuronal migration. Aberrant migration will lead to a range of human disorders including lissencephaly and subcortical band heterotopia [26], [27]. These conditions are always associated with cognitive deficits, motor impairment, dementia, and epilepsy [28]. In addition, neuronal migration occurs at the site of injury. It is also important to note that brain tumor cells can migrate long distances in the adult human brain. As we found that Nischarin is a key regulatory molecule that controls neuronal migration, it may have important physiological and pathophysiological implications for brain development, dementia, brain cancers and neurodegenerative disorders.

In summary, this work provides useful evidence that both Nischarin mRNA and protein are expressed in many regions and specific cells in the adult rodent brain, and play a role in the regulation of neuronal migration. Knowledge of the brain distribution of Nischarin presented in this paper may be a starting point for considering Nischarin as a potential therapeutic target for a broad spectrum of diseases of the nervous system.
